# Human atrial fibrillation and genetic defects in transient outward currents: mechanistic insights from multi-scale computational models

**DOI:** 10.1098/rstb.2022.0166

**Published:** 2023-06-19

**Authors:** Ghadah Alrabghi, Yizhou Liu, Wei Hu, Jules C. Hancox, Henggui Zhang

**Affiliations:** ^1^ Biological Physics Group, Department of Physics and Astronomy, University of Manchester, Manchester M13 9PL, UK; ^2^ Department of Physics, Faculty of Science, University of Jeddah, 21959 Jeddah, Saudi Arabia; ^3^ School of Physiology, Pharmacology and Neuroscience, Medical Sciences Building, University Walk, Bristol BS8 1TD, UK; ^4^ Key Laboratory of Medical Electrophysiology of Ministry of Education and Medical Electrophysiological Key Laboratory of Sichuan Province, Institute of Cardiovascular Research, Southwest Medical University, 646099 Luzhou, People's Republic of China

**Keywords:** atrial fibrillation, pro-arrhythmic effect, action potential, gain-of-function mutations, *I*_to_ channel, atrial model

## Abstract

Previous studies have linked dysfunctional *I*_to_ arising from mutations to *KCND3*-encoded Kv4.3 and *KCND2*-encoded Kv4.2 to atrial fibrillation. Using computational models, this study aimed to investigate the mechanisms underlying pro-arrhythmic effects of the gain-of-function Kv4.3 (T361S, A545P) and Kv4.2 (S447R) mutations. Wild-type and mutant *I*_to_ formulations were developed from and validated against experimental data and incorporated into the Colman *et al*. model of human atrial cells. Single-cell models were incorporated into one- (1D) and two-dimensional (2D) models of atrial tissue, and a three-dimensional (3D) realistic model of the human atria. The three gain-of-function mutations had similar, albeit quantitatively different, effects: shortening of the action potential duration; lowering the plateau membrane potential, abbreviating the effective refractory period (ERP) and the wavelength (WL) of atrial excitation at the tissue level. Restitution curves for the WL, the ERP and the conduction velocity were leftward shifted, facilitating the conduction of atrial excitation waves at high excitation rates. The mutations also increased lifespan and stationarity of re-entry in both 2D and 3D simulations, which further highlighted a mutation-induced increase in spatial dispersion of repolarization. Collectively, these changes account for pro-arrhythmic effects of these Kv4.3 and Kv4.2 mutations in facilitating AF.

This article is part of the theme issue ‘The heartbeat: its molecular basis and physiological mechanisms’.

## 1. Introduction

In the human myocardium, voltage-gated K*^+^* channel currents play major roles in the process of action potential (AP) repolarization [1,2]. One important potassium current is the Ca^2+^-independent transient outward K*^+^* channel current, *I*_to_, which rapidly activates and inactivates and underlies early (phase-1) AP repolarization. This sets the level of membrane potentials at the plateau phase of the AP, during which the calcium ions enter the intracellular space of cells via activated L-type Ca^2+^ channels. Alterations to *I*_to_ have been linked to several types of arrhythmia [3].

Previous studies have shown that increases in *I*_to_ are related to serious cardiac arrhythmias, such as lone atrial fibrillation (AF) [4,5], Brugada syndrome (BrS) [6,7], early repolarization syndrome [8,9] and J-wave syndrome [10,11]. Furthermore, alterations in *I*_to_ expression have been associated with other heart diseases, including hypertrophy, heart failure and sudden unexplained death syndrome [12–14]. A recent simulation study has also shown that altered *I*_to_ may strongly modulate AP duration, leading to an increased propensity of electrical alternans that predisposes to the onset of AF [15].

AF is one of the most common sustained cardiac arrhythmias [16–18]. It is characterized by rapid and irregular atrial excitations [19]. One basic mechanism underlying AF is re-entry, in which an excitation wave repeatedly re-excites the same region of cardiac tissue either by following a leading circuit substrate or in the form of spiral wave rotors, which might be facilitated and perpetuated by both electrical and structural remodelling [20].

Genetic defects producing dysfunctional K^+^ channels are also pro-arrhythmic [21–23]. With respect to *I*_to__,_ two gain-of-function mutations in the pore-forming channel subunit Kv4.3 encoded by *KCND3* have been reported in patients with early-onset of lone AF [4,5]. The first mutation (T361S) was identified in a proband with paroxysmal AF whose father was affected by persistent AF. *In vitro* electrophysiological studies have shown that this mutation alters *I*_to_ kinetics, manifested by a hyperpolarizing shift in both the steady-state activation and inactivation relations, and an increase in the current density by approximately 60% [4]. A preliminary simulation based on the Grandi *et al*. [24] model of the human atrial cell predicted abbreviation of action potential duration (APD) by the T361S mutation [4]. By contrast, the A545P Kv4.3 mutation, identified from genetic screening of a cohort of unrelated lone-AF patients, caused a slight hyperpolarization of the Kv4.3 steady-state inactivation relation. However, this mutation also increased Kv4.3 current density and slowed down the inactivation process, manifested by an increased inactivation time constant compared with that for the wild-type (WT) channel [5]. Preliminary simulations based on the Courtemanche *et al*. [25] model of human atrial cells (CRN) also predicted APD abbreviation. A third mutation (S447R) in *KCND2-*encoded Kv4.2 [26] has also been identified in patients with nocturnal paroxysmal AF. This mutation is different from T361S in Kv4.3, but shares some similarity to A545P, causing a slowing of channel inactivation, and exerting a gain-of-function effect. It has been hypothesized that S447R mutation may also shorten the atrial APD and be pro-arrhythmic, but this has not yet been demonstrated.

Although Kv4.3 and Kv4.2 mutations have been identified in AF patients, possible causality between these three identified genetic variants and AF has not been demonstrated, especially in respect of possible similarities or differences in their pro-arrhythmic effects, as the mutations occur in Kv4.3 (T361S and A545P) and Kv4.2 (S447R), two different proteins underlying the channels responsible for transient outward potassium current *I*_to_ [1].

Mathematical and computational modelling is valuable for the investigation of cardiac function in normal and pathophysiological conditions [27–29]. It also provides an alternative method for the interrogation of the functional consequences for cardiac electrophysiology of gene mutations, where appropriate genetically modified animal models are lacking. Therefore, this study implemented multi-scale computer modelling approaches to investigate a possible causative link between the three *I*_to_-associated mutations mentioned above and arrhythmogenesis of AF. In simulations, we investigated the functional impact of the three *I*_to_-associated gene mutations on human atrial electrical excitations and their conductions at the single-cell, tissue, and whole-organ levels in order to elucidate the mechanism(s) underlying the pro-arrhythmic effects of Kv4.2 and Kv4.3 mutations in AF.

## 2. Methods

### (a) *I*_to_ formulation and validation

**Figure 1.**
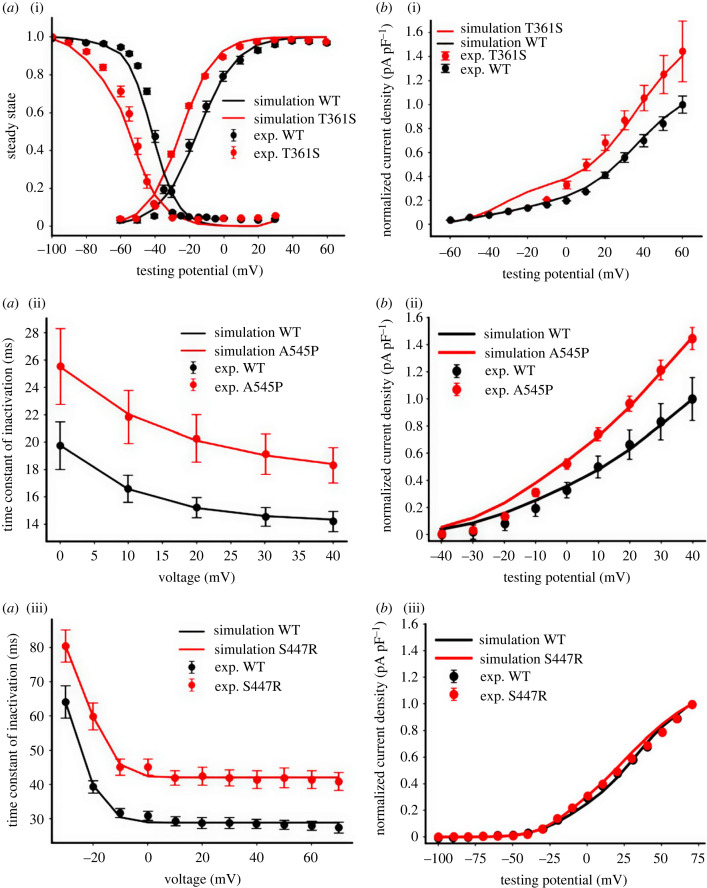
Simulation and experimental data on *I*_to_ kinetics and *I–V* relationship in WT and Kv4.3(T361S, A545P) and Kv4.2 (S447R) mutations. (*a*) (i) Steady-state activation and inactivation curves in WT and T361S mutation. (ii) Inactivation time constant in WT and A545P mutation. (iii) Inactivation time constant in WT and S447R mutation. (*b*) (i) *I–V* for WT and T361S mutation. (ii) *I–V* for WT and A545P mutation. (iii) *I–V* for WT and S447R mutation. Solid circles represent the experimental results (exp.) while the continuous lines represent the simulations. (Online version in colour.)

The Nygren *et al*. formulation [27] of *I*_to_ was implemented as the basal model, which was further updated based on the experimental data of patch-clamp study of *I*_to_ channel for the WT condition [4]. To obtain optimal parameters for the model, the Nelder–Mead simplex method [28] was used to best fit the simulation data to experimental data. The developed model equations and parameters were validated by their ability to reproduce the voltage-clamp experimental data of *I*_to_ kinetics for the WT channel recorded in HEK293 cells (figure 1). Simulation results from the updated *I*_to_ formulation were also validated by comparing simulations results with the recordings from previous studies [5,26].

Model parameters for the steady-state activation and inactivation variables and for the time constant of the inactivation were obtained by best fitting the simulation data to the corresponding experimental data from co-expression of

WT+KChIP2, mutant (MT) (Kv4.3)+KChIP2 or MT (Kv4.2) channels in cultured cells as shown, respectively, in figure 1*a*(i–iii) for the mutation conditions Kv4.3 T361S, Kv4.3 A545P and Kv4.2 S447R. For the T361S mutation, the time constants of the activation and inactivation remained the same as the WT model, but there was a significant hyperpolarizing shift to both steady-state activation and inactivation curves, as shown in figure 1*a*(i). For A545P and S447R mutations, there was an increase in the time constant of inactivation (figure 1*a*(ii,iii)), but the changes in the steady-state activation and inactivation curves were negligible. As the experimental data were obtained at room temperature (22°C), a temperature correction (*Q*_10_ = 3.5) was used when the formulations for the time constant were incorporated into the cell model, which was developed for body temperature (37°C) [25].

To best fit the simulated current–voltage (*I–V*) relationship to experimental data, the maximum channel conductance of *I*_to_ (*g*_to_) in T361S and A545P Kv4.3 mutations was increased, while in the S447R Kv4.2 mutation, it was slightly decreased relative to WT. As shown in figure 1*b*(i–iii), the simulated *I–V* curves in the three mutation conditions using the same voltage-clamp protocol as used experimentally matched experimental data, further validating the developed *I*_to_ channel model. As the AF patients in whom these mutations were identified were heterozygous for these missense mutations [4,5,26], owing to lack of experimental data we implemented a simple combination of 50% : 50% of WT and MT *I*_to_ to mimic the heterozygous condition of (Kv4.3) T361S and A545P mutations. For mimicking the heterozygous Kv4.2 S447R mutation, available experimental data on the inactivation time constant of *I*_to_ with co-expression of WT+Kv4.2 S447R mutation [26] were used to develop a heterozygotic *I*_to_ formulation (see electronic supplementary material, figure S1). Equations and equation parameters for WT and the three mutation conditions are listed in electronic supplementary material, table S1.

### (b) Single-cell model

The updated *I*_to_ equations were incorporated into the single-cell model of Colman *et al.* [29], in which the change in the membrane potential is given by

$$\frac{dV_{m}}{dt}=-\frac{I_{\mathrm{ion},\mathrm{tot}}}{C_{m}},$$

where *V*_m_ is the membrane potential, *I*_ion,tot_ the total ionic current including *I*_to_, and *C*_m_ the membrane capacitance.

The AP model was solved by using the forward explicit Euler method using a time step of 0.01 ms, while the Rush–Larsen method was used to solve equations for gating variables of ion channels [30]. Using the updated single-cell model, we quantified the functional impact of the three mutations on the characteristics of APs, the AP amplitude (APA), the resting membrane potential (RMP), the AP duration at 90 and 50% repolarization (APD_90_, APD_50_), the maximum upstroke velocity (MUV), the plateau potential and the APD restitution curves. Details of the methods for single-cell simulations are documented in the electronic supplementary material. In order to test whether our simulation results are model-independent, the Grandi *et al*. model of human atrial cells [24] was also used to quantify the functional impacts of the three mutations on the characteristics of APs. Results with that model are provided in the electronic supplementary material.

### (c) Tissue models

The single-cell atrial models for WT and MT conditions were incorporated into homogeneous one- (1D) and two-dimensional (2D) multicellular tissue models and a three-dimensional (3D) realistic anatomical model of the human atria. The monodomain equation for cardiac tissue models was used, which is given by the following partial differential equation [31]:

$$\frac{\partial V_{\;}}{\partial t}=\nabla \cdot D(\nabla V_{m})-\frac{I_{\mathrm{ion}}}{C_{m}},$$

where *D* is the diffusion tensor and ∇ is the 3D spatial gradient operator. To solve the equation, the explicit finite difference method with non-flux boundary conditions was used [31] to obtain numerical solutions of AP across the tissue.

The 1D atrial strand model of had a length of 25 mm, which was discretized into 100 nodes with a spatial resolution of 0.25 mm. The diffusion parameter *D* was set to the same value as used in our previous studies (0.21 mm^2^ ms^−1^), which produced a conduction velocity (CV) of 0.69 m s^−1^ along the atrial fibres, which is close to experimental data and previous studies [32–34].

Using the 1D tissue models, we computed the functional consequences of the three gene mutations on the tissue excitation properties, including the restitution curves of the CV, effective refractory period (ERP) and wavelength (WL) of excitation waves for the WT and the three mutation conditions. We also computed the rate dependence of atrial excitability (measured reciprocally by the excitation threshold of atrial tissue) in both WT and mutation conditions. Details of the methods for 1D simulations are documented in the electronic supplementary material.

#### (i) Tissue vulnerability

Cardiac tissue vulnerability was measured by the width of a temporal vulnerable window (VW), during which a premature stimulus (S2) applied to the refractory period of the cardiac tissue evoked a unidirectional conduction block that led to formation of re-entrant excitation waves underlying fibrillation [29,35,36]. The greater the VW width, the more vulnerable is the tissue to arrhythmogenesis. In this study, considering the regional difference in electrophysiological properties of atrial tissue at junctions of distinctive atrial tissue [37–39], we measured the VW width at two junctions, i.e. one between the crista terminalis and pectinate muscles (CT/PM) and the other between the left atrium and pulmonary veins (LA/PV). Details for computing VW are documented in the electronic supplementary material.

#### (ii) Two-dimensional simulation of re-entrant excitation wave

Dynamic behaviours of re-entrant excitation waves were investigated using an idealized 2D sheet of isotropic tissue with the dimensions 100 × 100 mm^2^, which was discretized by a spatial resolution of 0.25 mm, forming a grid of 400 × 400 nodes, each of which was simulated by the right atrial cell model. In simulations, the functional impacts of the three gene mutations on the lifespan, core meandering pattern and dominant frequency (DF) of re-entrant excitation waves were investigated using the methods described in the electronic supplementary material.

#### (iii) Three-dimensional anatomical model

The human atria have 3D anatomical structures that are spatially heterogeneous in electrophysiological properties and anisotropic in atrial fibre spatial arrangements and intercellular electrical coupling [29]. To test whether the results obtained at the 2D level reflected those in the 3D model, the realistic 3D human atrial anatomical model developed in our previous study [29] was used to investigate the effects of *I*_to_ mutations on the dynamic behaviours of re-entrant excitation waves (scroll waves), including their lifespan. Details about 3D simulations are documented in the electronic supplementary material.

## 3. Results

### (a) Effects of *I*_to_ mutations on the action potential and action potential duration restitution properties

**Figure 2.**
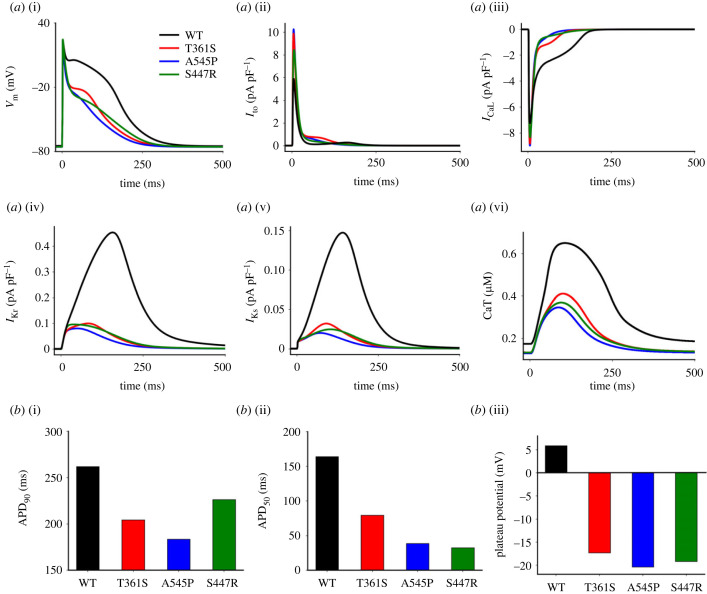
Effects of gain-of-function of *I*_to_ mutations to Kv4.3 (T631S and A545P) and Kv4.2 (S447R) on the action potentials (APs) elicited by using the right atrial cell model. (*a*) (i) Time course of APs at 1000 BCL. (ii)–(vi) Corresponding time courses of *I*_to_, *I*_CaL_, *I*_Ks_, *I*_Kr_ and calcium transient (CaT) during AP. (*b*) (i) Action potential duration (APD) measured at 90% repolarization (APD_90_). (ii) APD measured at 50% repolarization (APD_50_). (iii) Plateau potential (measured as the mean of membrane potential during the plateau phase of the AP). (Online version in colour.)

We first investigated the functional impact of the mutations on atrial AP profile. Figure 2 shows simulation results from the right atrial cell model for illustration. As shown in figure 2, all mutations caused marked early phase-1 repolarization, leading to a depressed plateau potential and accelerated repolarization compared with the WT condition (figure 2*a*(i)). Consequently, the APDs and ERPs (see electronic supplementary material, table S2) were reduced in the three mutation conditions. The measured APD_90_ were 204.4, 183.6 and 226.2 ms for (Kv4.3) T361S, A545P and (Kv4.2) S447R, respectively, which were markedly shorter than 261.9 ms for the WT. The corresponding ERPs were 123.2, 113.2 and 150.4 ms, respectively for the (Kv4.3) T361S, A545P and (Kv4.2) S447R conditions as compared with 205.1 ms for the WT. Such abbreviated APs were associated with an augmented magnitude of *I*_to_ during the time courses of APs in the mutation condition (figure 2*a*(ii)). In the heterozygous mutation conditions, MT *I*_to_ also caused changes in the AP profiles, leading to abbreviated ERPs (see electronic supplementary material, figure S3 and table S2). The measured ERPs at basic cycle length (BCL) = 800 ms were 177.1, 192.2 and 178.0 ms for WT+(Kv4.3) T361S, WT+(Kv4.3) A545P and WT+(Kv4.2) S447R, respectively, which were shorter than 205.1 ms for the WT condition. As the effects of the heterozygous mutations on ERP reduction at fastpacing rates (e.g. BCL < 500 ms; as seen in AF) were qualitatively similar to those of their corresponding homozygous mutations, the analyses that follow focused on homozygous mutation settings. Simulation results from the Grandi *et al*. model showed similar effects of the three gene mutations in APD abbreviation of the human atrial cell model. Results are presented in the electronic supplementary material, figure S8 and table S3.

We further investigated how the mutations caused APD reduction. It was shown that the mutation-depressed AP plateau potential reduced the magnitude of *I*_CaL_ during the plateau, though its peak amplitude was increased (figure 2*a*(iii)) owing to a more hyperpolarized resting potential together with a rapid decrease in phase-1 membrane potential which increased *I*_CaL_ activation (figure 2*a*(i)). Such a reduced *I*_CaL_ provided insufficient inward depolarizing current to counterbalance outward repolarizing *I*_Kr_ and *I*_Ks_ to maintain the plateau phase, though the magnitudes of *I*_Kr_ and *I*_Ks_ during the AP course were also reduced owing to a secondary effect of a depressed plateau potential (figure 2*a*(iv,v)). As such, a rapid completion of repolarization course was established, leading to APD reduction. A shortened AP time course and reduced *I*_CaL_ also led to decreased intracellular Ca^2+^ concentrations (figure 2*a*(vi)), resulting from a reduced total Ca^2+^ influx from *I*_CaL_ and Ca^2+^ release from the sarcoplasmic reticulum (SR), which may reduce the active contractile force and thus impair the mechanical contraction of the heart (examination of mechanical consequences was beyond the intended scope of this study).

The three *I*_to_-associated mutations also changed other characteristics of APs. A quantitative summary of the effects of these mutations on the characteristics of APs computed from the atrial cell model is presented in table 1. The mutations caused a slightly increased APA, and a hyperpolarized RMP by about 1 mV. They increased MUV noticeably (e.g. the measured MUV was 196.5 V s^−1^ in the Kv4.3 A545P mutation, greater than 188.3 V s^−1^ in the WT). The measured APDs at both 90 (APD_90_) and 50% (APD_50_) repolarization, as well as the plateau potentials in WT and mutations are shown in figure 2*b*(i–iii), respectively.

**Table 1.** Summary of changes in action potential characteristics by Kv4.3 (T361S, A545P) and Kv4.2 (S447R) mutations.

	APA (mV)	RMP (mV)	APD_90_ (ms)	APD_50_ (ms)	MUV (V s^−1^)	plateau potential (mV)
WT	98.7	−74.6	261.9	164.1	188.3	5.9
T361S	99.5	−75.5	204.4	79.6	195.7	−17.3
A545P	99.8	−75.6	183.6	38.8	196.5	−20.4
S447R	100	−75.5	226.2	32.5	195.6	−19.2

**Figure 3.**
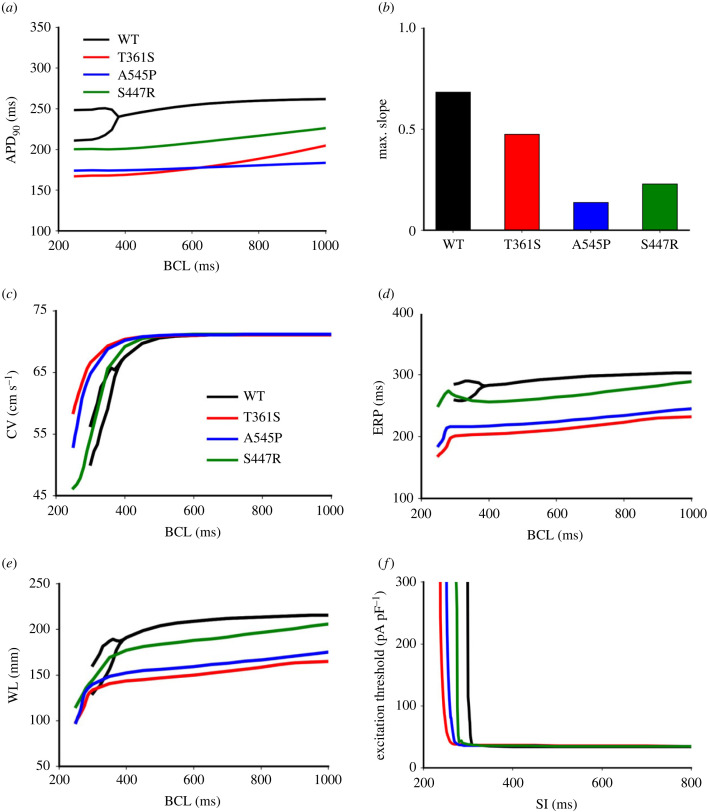
Effects of gain-of-function of *I*_to_ mutations Kv4.3 (T631S and A545P) and Kv4.2 (S447R) on atrial cell and tissue restitution properties. (*a*) Action potential duration (APD) restitution. (*b*) Maximal slope of the APD restitution curves. (*c*) Conduction velocity. (*d*) Effective refractory period (ERP). (*e*) Wavelength (WL). (*f*) Excitation threshold against S1–S2 stimulus time intervals.

Effects of the three mutations on atrial APD restitution are shown in figure 3*a*. At large BCLs (i.e. at slow pacing rates), the measured APD_90_ was smaller with mutations than in the WT condition. However, at small BCLs (i.e. at fast pacing rates), there was a leftward shift of the APD restitution curves, implying the mutation favoured atrial excitations at high rates, which were often observed in AF. Note that, in the WT condition, a noticeable bifurcation in the APD restitution curve was observed, suggesting the genesis of AP alternans. However, for the mutations, the bifurcation disappeared except in the case of Kv4.2 S447R, in which a small bifurcation in the APD restitution curve was observed (figure 3*a*). The disappearance of AP alternans was attributable to the abbreviated APD. In addition, the mutations flattened APD restitution curves, implying a loss of rate adaptation of atrial cell excitation. The computed maximum slope values for the APD restitution curves were 0.477, 0.139 and 0.212, for (Kv4.3) T361S, A545P and (Kv4.2) S447R, respectively, which were markedly smaller than 0.684 for the WT (figure 3*b*). Loss of rate adaptation of atrial excitation is believed to be pro-arrhythmic [40].

### (b) Effects of *I*_to_ mutations on conduction restitution properties

Using the 1D strand model, we investigated the functional consequences of the three gene mutations for the restitution properties of atrial CV (restitution curve), ERP (restitution curve) and WL of excitations (WL restitution curve). We also investigated their effects on the excitability of atrial tissue, as measured reciprocally by the computed excitation threshold. Figure 3*c* shows the computed CV restitution curve. At large BCLs (i.e. at slow pacing rates), there were no noticeable differences in the computed CVs between WT and mutation conditions. However, at small BCLs (i.e. at fast pacing rates), the conduction of atrial excitation failed in WT, but was maintained in the mutation conditions. The mutations caused a leftward shift to the CV restitution curves as compared with that of WT, further demonstrating the mutations favoured for conduction of rapid atrial excitation. A similar leftward shift to the ERP restitution (figure 3*d*) and the WL restitution (figure 3*e*) curves was observed, suggesting that the favoured atrial conduction at rapid pacing rates was attributable to the reduced ERP of the tissue, which reduced WL, facilitating excitability at short time intervals following a previous excitation.

A leftward shift in the rate-dependent excitation threshold of atrial cells (a reciprocal measure of cell excitability) was also observed. Results are shown in figure 3*f*. In the WT condition, with a decrease in the stimulus time interval (SI) from 1000 to 350 ms, there was non-noticeable change in the computed excitation threshold. However, with a further decrease of SI to less than 350 ms, there was a sharp increase in the computed excitation threshold owing to the SI falling into the ERP of atrial excitations, during which tissue excitability was reduced. At a critical value of SI = 308 ms, the measured threshold became extremely large, indicating the loss of atrial excitability. In the three mutation conditions, the measured excitation threshold at SI greater than 350 ms was slightly greater than that for the WT condition, owing to a more hyperpolarized resting potential (table 1), which required a greater stimulus amplitude for the membrane potential to reach the upstroke potential. However, with SI less than 350 ms, the measured excitation threshold is much less than that of the WT, owing to abbreviated APD/ERP. The measured critical values of SI at which atrial tissue lost excitability were 259, 272 and 287 ms for (Kv4.3) T361S, A545P and (Kv4.2) S447R mutations, respectively, which were markedly shorter than 308 ms in the WT condition, indicating the mutations increased atrial tissue's excitability at high excitation rates.

**Figure 4.**
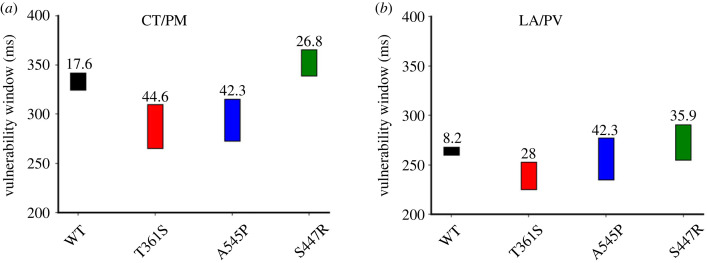
Effects of gain-of-functions of *I*_to_ mutations (Kv4.3) T631S and A545P and (Kv4.2) S447R on the width of the temporal vulnerable window (VW) during which unidirectional conduction block was observed in response to a premature stimulus. (*a*) VWs measured at the CT/PM junction. (*b*) VWs measured at the LA/PV junction. (Online version in colour.)

Figure 4 shows the computed width of VW at atrial junctions of CT/PM and LA/PV, characterizing the susceptibility of atrial tissue to arrhythmogenesis. It was shown that the mutations increased the width of VW. At the CT/PM junction, the measured width of VW increased from 17.6 ms in the WT condition to 44.6*,* 42.3 and 26.8 ms for (Kv4.3) T361S, A545P and (Kv4.2) S447R mutations, respectively (figure 4*a*). Though the effect of the three mutations on VW was qualitatively similar, there was a quantitative difference among them, with the T361S causing the largest increase in the width of VW. At the LA/PV junction, the mutations increased the width of VW from 8.2 ms in WT to 28, 42.3 and 35.9 ms, respectively, for (Kv4.3) T361S, A545P and (Kv4.2) S447R mutations (figure 4*b*), albeit in a scenario without consideration of relative contribution of different Kv4.*x* isoforms to *I*_to_ [3,41] (see Discussion). Collectively, these results suggested that the atrial tissue was more vulnerable to arrhythmogenesis owing to the *I*_to_-associated mutations.

### (c) Effects of *I*_to_ mutations on spiral wave dynamics

**Figure 5.**
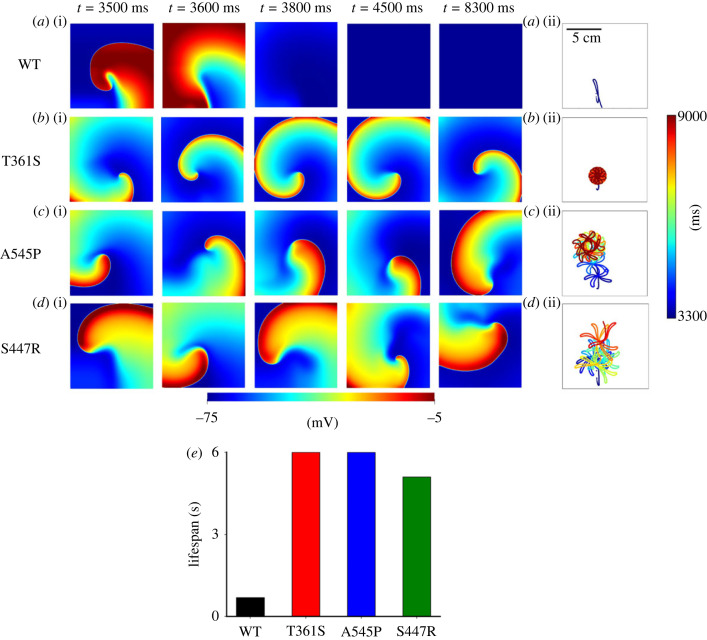
Snapshots of two-dimensional simulated re-entrant spiral waves at different timings and their corresponding meandering pattern of the core in WT (*a*(i,ii)), T361S Kv4.3 (*b*(i,ii)), A545P Kv4.3 (*c*(i,ii)) and S447R Kv4.3 (*d*(i,ii)) conditions. The corresponding lifespans of re-entrant spiral waves for different conditions are shown in (*e*). (Online version in colour.)

Figure 5 shows the functional impacts of the three gain-of-function mutations on modulating the dynamical behaviours of re-entrant excitation waves using an idealized 2D tissue sheet model.

In the WT case, the simulated re-entrant spiral waves lasted approximately 690 ms before self-termination, as shown by the snapshots of re-entrant spiral waves at different timings, as shown in figure 5*a*(i). Such short-lasting spiral waves were associated with a non-stationary drifting of the spiral wave core, which followed a linear path excursion (figure 5*a*(i,ii)). As such, the spiral wave self-terminated when its core meandered out of the tissue boundary.

However, in the case of the Kv4.3 and Kv4.2 mutations, the spiral waves became more sustained. In the case of the Kv4.3 T361S (figure 5*b*(i)) and Kv4.3 A545P (figure 5*c*(i)) mutations, spiral waves were sustained through the whole period of simulations (6 s), and their cores followed flower-like or hyper-meandering patterns, indicating more stationary and stable rotations [42,43]. After a period of transition, such hyper-meandering settled down to a constrained small area, leading to sustained re-entry (figure 5*b*(ii), *c*(ii)). In the Kv4.2 S447R condition (figure 5*d*(i)), the spiral wave lasted about 5.1 s*.* Its core followed a more complicated transient path, which was then constrained to a local area (figure 5*d*(ii)). The measured lifespan (LS) and DF of simulated spiral waves are shown in table 2, clearly indicating that the mutations increased the persistence of re-entry.

**Table 2.** Dominant frequency (DF) and lifespan (LS) of re-entrant spiral waves in two-dimensional model of the human atria for WT and (Kv4.3) T361S, A545P and (Kv4.2) S447R mutation conditions.

	WT	T361S	A545P	S447R
DF (Hz)	N/A	5.8	4.1	3.4
LS (s)	0.69	6	6	5.1

### (d) Effects of *I*_to_ mutations on scroll wave dynamics

**Figure 6.**
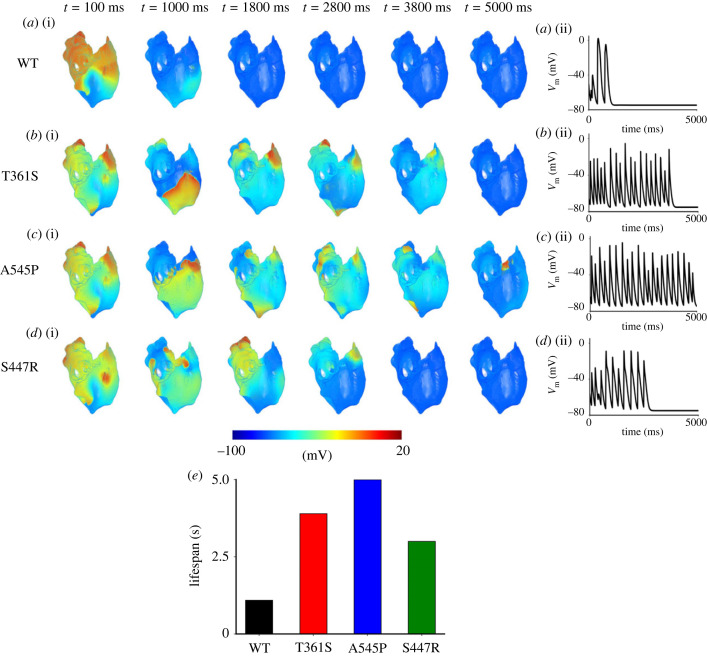
Snapshots of three-dimensional simulated scroll waves at various timings and the corresponding AP time series recorded from a local point in the right atrium in WT (*a*(i), *a*(ii)), T361S Kv4.3 (*b*(i), *b*(ii)), A545P Kv4.3 (*c*(i), *c*(ii)) and S447R Kv4.2 (*d*(i), *d*(ii)) conditions. The computed lifespans for WT and mutation conditions are shown in (*e*). (Online version in colour.)

To test if the simulation results from a 2D idealized atrial model are representative of the 3D human atria with detailed anatomical structures and heterogeneous electrophysiological properties, further simulations were conducted using a 3D model of the human atria [29]. Results are shown in figure 6 for the simulated re-entrant scroll waves. In the figure, snapshots of excitation waves for different timings for WT (figure 6*a*(i)), Kv4.3 T361S (figure 6*b*(i)), Kv4.3 A545P (figure 6*c*(i)) and Kv4.2 S447R (figure 6*d*(i)) are shown, together with their corresponding time traces of recorded APs from a local point of the right atrial tissue (figure 6*a*(ii)–*d*(ii)). It was shown that in the WT condition, the scroll waves were sustained only for approximately 1.1 s, before self-termination. The three mutations increased the lifespan of the scroll waves and their DF. In the case of Kv4.2 S447R and Kv4.3 T361S mutation conditions, the LS of the scroll waves was increased to approximately 3 and 3.9 s, respectively (table 3). In the case of the Kv4.3 A545P mutation condition, the simulated scroll wave lasted through the whole simulation period of 5 s. The measured LSs for WT and the three mutations are presented in figure 6*e*. The measured DF was 2.7 Hz for WT, which increased to 5.2, 5.5 and 3.5 Hz for Kv4.3 T361S, Kv4.3 A545P and Kv4.2 S447R mutation conditions, respectively. The results from the 3D model corroborated those from 2D models, further demonstrating the increased capability of atrial tissue to sustain cardiac arrhythmia in the mutation conditions.

**Table 3.** Dominant frequency (DF) and lifespan (LS) of re-entrant scroll waves in three-dimensional model of the human atria for WT and Kv4.3 (T361S, A545P) and Kv4.2 (S447R) mutation conditions.

	WT	T361S	A545P	S447R
DF (Hz)	2.7	5.2	5.5	3.5
LS (s)	1.1	3.9	5	3

## 4. Discussion

### (a) Main findings

In this study, we implemented multi-scale computer simulation approaches to investigate the functional consequences of three gene mutations (T361S and A545P in Kv4.3; S447R in Kv4.2), specifically on atrial electrical excitation and conduction. Our major findings are (i) at the single-cell level, the augmented *I*_to_ arising from the mutations accelerated atrial repolarization, and resulted in a decreased APD and ERP. It flattened the APD restitution curve, suggesting a loss of APD rate adaptation. It also impaired intracellular Ca^2+^ cycling, resulting in a reduced amplitude of the intracellular Ca^2+^ transient. In the heterozygous mutation conditions, the augmented *I*_to_ also changed the AP profile, leading to abbreviation of ERP which was more marked at faster pacing rates (e.g. BCL less than 500 ms; as seen in AF conditions); (ii) at the tissue level, the mutations decreased the excitation WL. They also caused leftward shifts in the restitution curves of CV, WL and ERP, favouring atrial conduction at high rates that cannot be conducted in WT tissue. The mutations also modulated atrial excitability and increased vulnerability of tissue to unidirectional conduction block; (iii) the mutations affected the dynamic behaviours of re-entry by modulating their meandering pattern to favour sustained re-entry, prolonging the lifespan of re-entry; (iv) results from the 3D model corroborated those from 2D simulations, further demonstrating the impact of the mutations on atrial electrical excitations. Collectively, these findings provide novel mechanistic insights into understanding the pro-arrhythmic effects of an augmented *I*_to_ by the three gain-of-function mutations.

### (b) Mechanisms for pro-arrhythmic effects of *I*_to_ gain-of-function mutations

Our simulation results have shown that the pro-arrhythmic effect of an augmented *I*_to_ due to the gain-of-function mutations of Kv4.3 (T361S, A545P) and Kv4.2 (S447R) was reflected by two main factors: one is an increased capability of tissue to sustain re-entry; the other is an increased vulnerability to initiate re-entry. Here, we analysed possible ionic mechanisms underlying the two pro-arrhythmic effects of the *I*_to_ mutations.

#### (i) Mechanisms for sustained re-entry

The increased ability of tissue to sustain re-entry in the settings of the mutations studied here is attributable to a shortened excitation WL. This in turn arises owing to abbreviated cellular APD consequent on accelerated repolarization. In our simulations, this led to a loss of APD rate adaptation at the cellular level and reduced WL at the tissue level. As such, re-entrant excitation waves in MT tissue required a smaller substrate size to rotate and evolve as compared with WT tissue, allowing them to persist. In addition, the mutations modulated the dynamic behaviours of the re-entry by altering the pattern of meandering, which also helps to sustain re-entry. In the WT condition, the re-entry followed a linear meandering path and self-terminated when it meandered out of the tissue's boundary. However, in the mutation conditions, the re-entry first followed curved petal paths, which were then constrained to a localized region. As such, stationary/stable re-entry was established. Such increased stationarity of re-entry in the MT tissue was attributable to the shortened ERP and WL, and the loss of their rate dependence (i.e. the restitution properties). In the WT condition, owing to large WLs of excitation waves, the succeeding wavefront ran into the refractory tail region of its preceding wave, where tissues were not yet completely recovered from their previous excitation, leaving no space for the wavefront to enter. Therefore, the wavefront near the core drifted (meandered) away from its current position. In the mutation conditions, owing to reduced WL and ERP, there was a sufficient gap between the succeeding wavefront and the refractory tail of the preceding wave, permitting the core to wander less as compared with the WT condition. As such, a stationary re-entry was established.

#### (ii) Ionic basis for action potential duration and effective refractory period abbreviation

It is of interest to understand how an augmented *I*_to_ contributed to APD and ERP reduction. *I*_to_ provides an outward current contributing to the repolarization of APs. However, it is different from other types of K^+^ currents, such as *I*_Kr_ and *I*_Ks_, which modulate the late/final phase of AP repolarization and have a direct impact on APD. Owing to its fast activation and rapid inactivation kinetics, *I*_to_ contributes primarily to the early phase of repolarization, producing a notch in the phase-1 of the AP.

In the present study, we have shown that an increased *I*_to_ caused a rapid and dramatic phase-1 repolarization that led to a depressed plateau potential to well below 0 mV (at about −20 mV), far from the membrane potential at which maximal *I*_CaL_ was activated. Consequently, a significant reduction in the sustained component of *I*_CaL_ after a brief increase of its peak amplitude was observed, which could not counterbalance *I*_Kr_ and *I*_Ks_ to maintain phase-2 plateau potential, though the magnitudes of *I*_Kr_ and *I*_Ks_ were also reduced secondarily owing to the depressed plateau potential (figure 2). The greater attenuation of sustained *I*_CaL_ than of *I*_Kr_ and *I*_Ks_ led to accelerated AP repolarization, resulting in reduced APD/ERP. Thus, the APD shortening by an augmented *I*_to_ was attributable to *I*_CaL_ reduction, resulting secondarily from a depressed plateau potential. Note that APD reduction by an augmented *I*_to_ arising from the gain-of-function mutations of Kv4.3 (T361S, A545P) and Kv4.2 (S447R) was replicated in simulations using the Grandi *et al*. model (electronic supplementary material, figure S8). This observation agreed with the study of Sutanto in showing that an augmented *I*_to_ abbreviated APD of atrial myocytes consistently across different computational models of atrial cardiomyocytes [44].

#### (iii) Increased tissue vulnerability for arrhythmogenesis

The measured width of the VW at major atrial tissue junctions of PM/CT and PV/LA was greater in MT tissue models, indicating an increased risk of arrhythmogenesis [45,46]. The increase in the width of the VW in the MT tissue was attributable to the amplified APD dispersion at the junctions by the three mutations. Owing to heterogeneous electrophysiological properties of the atrial tissue [29,37,47], there is a marked intrinsic APD/ERP dispersion in atrial tissue including major junctions. As such, there is a time window, after which cells with shorter APDs have recovered from their preceding excitation, while cells with longer APDs have not. When a premature excitation occurs during the time window, the evoked excitation wave only propagates in the regions/directions of tissue with shorter APD but are blocked in the regions/directions with longer APD, leading to directional conduction block that facilitates re-entry.

In this study, altered APD dispersion in atrial tissue by the three mutations was observed. For example, the measured APD difference between PM and CT cells was about 50.6 ms in the WT condition. However, it became 116.6, 122.3 and 131.6 ms for the (Kv4.3) T361S, A545P and (Kv4.2) S447R mutation conditions, respectively.

### (c) Relevance to previous studies

Altered *I*_to_ with either increased or decreased current density arising from gene mutations or disease-induced electrical remodelling has been identified in patients with AF [4,5,26,48]. It has been postulated that dysfunctional *I*_to_ may produce abnormal repolarization, leading to cardiac arrhythmogenesis.

Previous studies have linked accelerated repolarization of APs to increases in several voltage-gated K*^+^* channel currents in AF [19,41,49]. Mechanistically, these augmented potassium currents (*I*_Ks_*, I*_Kr_*, I*_Kur_ *and I*_K1_) may lead to abbreviated APD/ERP, and reduced CV and tissue excitability, leading to shortened WL, favouring initiation and maintenance of re-entry [34,50–52]. In the present study, we showed that an augmented *I*_to_ has a similar impact on APD abbreviation, like other augmented potassium channel currents, but via a different mechanistic pathway. Instead of speeding up the late and final phase of AP repolarization as would occur by an augmented *I*_Ks_*, I*_Kr_*, I*_Kur_ or *I*_K1_, an augmented *I*_to_*-*abbreviated APD occurred via a reduced sustained component of *I*_CaL_ owing to a depressed plateau potential. This result added new understanding of potential integral and coordinated actions of *I*_to_ with other ion channels in the process of AP repolarization.

Prior preliminary computer simulations have shown that an augmented *I*_to_ arising from Kv4.3 T361S and A545P mutations reduced atrial APDs [4,5]. This is consistent with the observation of the present study. However, these studies did not investigate potential mechanisms underlying APD reduction, and consequent pro-arrhythmic effects of the mutations. The results presented in this study therefore extend these earlier findings; they show the pro-arrhythmic effect of the (Kv4.3) T361S, A545P and (Kv4.2) S447R mutations in association with the loss of rate adaptation of atrial APD_90_ restitution. Owing to flattened APD_90_ restitution curves, re-entry in the MT tissue models was more stationary/stable, leading to sustained re-entry with longer LS. Similar observations on the pro-arrhythmic effects of the loss of rate adaptation of cardiac excitation have been reported in previous studies [34,53,54].

Our findings on the pro-arrhythmic effects of augmented *I*_to_ may also shed light on understanding of other *I*_to_-related cardiac arrhythmias, such as BrS [6,7] and J-wave syndrome [10,11], which warrant future studies.

## 5. Limitations

Limitations of the basal model of the human atrial cells have been discussed in detail elsewhere [25,29]. In the present study, there are some other potential limitations. One concerns the formulation of *I*_to_ used in this study. Owing to the lack of experimental data from human atrial cells, the basal formulation of *I*_to_ was based on data from rabbit atrial myocytes [27], and may be different from that in human atrial cells owing to species differences [50,55]. In the development of models for WT and MT conditions, the experimental data used to fit the kinetics of *I*_to_ were not from native cardiac cells, but from recordings in cultured HEK293 (human embryonic kidney 293) (Kv4.3 T361S and Kv4.2 S447R) or CHO-K1 (Chinese hamster ovary) (Kv4.3 A545P) cells with WT or MT channels co-expressed with the auxiliary subunit K^+^ channel-interacting protein (KChIP2) [4,5,26]. Therefore, the kinetics of *I*_to_ from heterologous expression experiments may differ from those in native cardiac myocytes, in which interactions with further accessory subunits may occur. In the WT model, the density of *I*_to_ was the same as used in the basal model of human atrial cells [25,32]; however, the use of this value may need to be updated when more data from native human atrial cells become available as the levels of *I*_to_ density could have an impact on modulation of cellular APD [44]. In addition, the model for *I*_to_ is a general model, without considering specific kinetics of *I*_to_ components encoded by different isoforms (Kv1.4, Kv4.2 and Kv4.3) [55–57], nor different current amplitude owing to their different protein expression levels in cardiac tissue, though Kv4.3 expression dominates in human atrial tissues [3,49]. A study of regional ion channel transcript expression in the undiseased human heart has reported comparatively low expression of Kv1.4 and Kv4.2 in (right) atrium compared with the expression of Kv4.3 [58]. Moreover, patch-clamp recording from human atrial myocytes is consistent with little contribution of Kv1.4 to atrial *I*_to_ (60). It is therefore likely that simulated changes to *I*_to_ based on mutation-induced alterations to Kv4.3 current alone predict fairly well mutation-induced changes to native atrial *I*_to_. However, it is also possible that simulated changes to *I*_to_ based on Kv4.2 current alone may overestimate the S447R mutation's effects, given that Kv4.3 normally predominates. Future interrogation of this issue would require an *in silico* recapitulation of *I*_to_, taking into account relative contributions of Kv4.3 and Kv4.2 [58]. AF patients in which these mutations were identified were heterozygous for each mutation [4,5,26]. Here, however, owing to lack of or incomplete experimental data (Kv4.3 T361S, Kv4.3 A545P and Kv4.2 S447R), MT *I*_to_ formulations were based on experimental data recorded from homozygous expression of the MT channel, not co-expression with WT to mimic patient heterozygosity except for Kv4.2 S447R. In a limited number of (single-cell) simulations, a simple and idealized case of heterozygous mutations by using a combination of 50% : 50% WT and MT *I*_to_ for heterozygosity (Kv4.3 T361S, Kv4.3 A545P) or experimental data-based heterozygous *I*_to_ mutation (Kv4.2 S447R) were considered and these simulations highlighted reduction of ERP in the heterozygous state, particularly at faster pacing rates (shorter cycle lengths). However, further investigations with improved *I*_to_ formulations and consideration of different subunits based on both homozygous and heterozygous expression of WT and MT subunits are needed when more experimental data become available. Another potential limitation concerns the use of tissue models. In the 2D model, we assumed homogeneous atrial electrical properties, lacking considerations of atrial intrinsic electrophysiological properties. However, in the 3D model, we considered detailed anatomical structures, and heterogeneous electrophysiological and anisotropic properties of atrial tissue. We did not include potential remodelling of atrial electrical properties, intercellular gap junctional coupling or fibrosis populations, each of which may play an important role in arrhythmia genesis. In addition, we did not consider the mechanical contraction of the atria, which could also have a potential contribution to the initiation and maintenance of AF [58,59]. While we make these potential limitations explicit, they do not alter our conclusions on the mechanisms underlying the pro-arrhythmic effects of increased *I*_to_ arising from the three gain-of-function mutations.

## 6. Conclusion

In this study, we used the Colman *et al*. human atrial model to investigate the pro-arrhythmic effects of an augmented *I*_to_ in human atrial tissue, arising from three gain-of-function mutations. We found that an augmented *I*_to_ accelerated the atrial repolarization process, leading to abbreviated APD via a secondary effect of a reduced *I*_CaL_ due to a depressed AP plateau potential. It caused the loss of rate adaptation of APD, ERP and WL, which contributed to tissue's increased ability to sustain re-entry. Our simulations also showed that the mutations increased tissue susceptibility to the initiation of re-entry at major atrial junctions, owing to augmented APD dispersion. These findings establish a causal link between the *I*_to_ mutations and AF, providing novel mechanistic insights into understanding how impaired AP repolarization arising from dysfunctional *I*_to_ increases the risk of atrial arrhythmias.

## Data Availability

Data are all included in the manuscript and online supplementary material. Source codes for human atrial cells in WT and MT conditions are available on request to the corresponding author at henggui.zhang@manchester.ac.uk.
